# 

**Published:** 1896-01-18

**Authors:** 


### CONTENTS.

The Physiology of Fighting - 259

Surgical Fees at Charity Hospitals - 260

On Modern Progress in Ophthalmic Medicine and Surgery. By R. B. CARTER, M.D.—Squint - 261

Medical Progress and Hospital Clinics—

Sleep and its Disorders.—II. By Tom Taylor, M.D. - 265

PROGRESS IN MEDICINE.—Diseases of the Lungs - 266

PROGRESS IN SURGERY.—Rectal Surgery - 268

Surgery of the Spine - 269

THERAPEUTIC NOTES - 269

NEW APPLIANCES AND THINGS MEDICAL - 270

Modern Sociology.—The Wastrels of Modern Cities - 262

Annotations.—A Latent Danger in Chlorosis—The Fate of the Microbe—Stuffy Railway Carriages - 263

Notes and News - 264

The Institutional Workshop—

PRACTICAL DEPARTMENTS—The "Gorham" Bed - 271

THE STORY OF THE INSANE FROM YEAR TO YEAR - 271

BOSTON HOSPITAL TENT SERVICE - 272

Editor's Letter Box - 272

Scraps and Gleanings - 272

The Book World of Medicine and Science - 273

### EXTRA SUPPLEMENT.—THE NURSING MIRROR.

News from the Nursing World.—Nurses at Windsor—A Vacancy at East Grinstead—District Work at Plaistow—Our Needlework Competition—Correspondence Classes—Warwick District Nursing—Former Patients Entertained—Fire at Mullingar Asylum—Women on Hospital Boards—The Wounded in Africa—Provision in Sickness—Our Sister in India—Progress in the Colonies—Registration of Nurses—Short Items - cxxvii

Lectures on Nursing. V.—The Administration of Medicine - cxxix

Royal British Nurses' Association - cxxx

Royal London Ophthalmic Hospital, Moorfields - cxxx

The Registration of Nurses - cxxxi

Queen Victoria Jubilee Institute for Nurses - cxxxii

Our American Letter - cxxxiii

The Winter Exhibitions - cxxxiv

Entertainments - cxxxiv

Dr. Barnardo's Entertainment at the Albert Hall - cxxxv

Lectures to Nurses - cxxxv

Everybody's Opinion - cxxxv

Notes and Queries - cxxxvi

For Reading to the Sick - cxxxvi

Appointments—Minor Appointments - cxxxvi

